# Metabolic Reprograming of Macrophages: A New Direction in Traditional Chinese Medicine for Treating Liver Failure

**DOI:** 10.1155/jimr/5891381

**Published:** 2024-12-24

**Authors:** Junli Zhang, Na Li, Xiaoyu Hu

**Affiliations:** ^1^Department of Infectious Diseases, Hospital of Chengdu University of Traditional Chinese Medicine, Chengdu, China; ^2^Department of Clinical Medicine, Chengdu University of Traditional Chinese Medicine, Chengdu, China

**Keywords:** acute liver failure, Chinese medicine, glucose metabolism, macrophage polarization, signal transduction

## Abstract

Acute liver failure (ALF) is a fulminant clinical syndrome that usually leads to multiple organ failure and high mortality. Macrophages play a crucial role in the initiation, development, and recovery of ALF. Targeting macrophages through immunotherapy holds significant promise as a therapeutic strategy. These cells exhibit remarkable plasticity, enabling them to differentiate into various subtypes based on changes in their surrounding microenvironment. M1-type macrophages are associated with a pro-inflammatory phenotype and primarily rely predominantly on glycolysis. In contrast, M2-type macrophages, which are characterized by anti-inflammatory phenotype, predominantly obtain their energy from oxidative phosphorylation (OXPHOS) and fatty acid oxidation (FAO). Shifting macrophage metabolism from glycolysis to OXPHOS inhibits M1 macrophage activation and promotes M2 macrophage activation, thereby exerting anti-inflammatory and reparative effects. This study elucidates the relationship between macrophage activation and glucose metabolism reprograming from an immunometabolism perspective. A comprehensive literature review revealed that several signaling pathways may regulate macrophage polarization through energy metabolism, including phosphatidyl-inositol 3-kinase/protein kinase B (PI3K/AKT), mammalian target of rapamycin (mTOR)/hypoxia-inducible factor 1*α* (HIF-1*α*), nuclear factor-*κ*B (NF-*κ*B), and AMP-activated protein kinase (AMPK), which exhibit crosstalk with one another. Additionally, we systematically reviewed several traditional Chinese medicine (TCM) monomers that can modulate glucose metabolism reprograming and influence the polarization states of M1 and M2 macrophages. This review aimed to provide valuable insights that could contribute to the development of new therapies or drugs for ALF.

## 1. Introduction

Acute liver failure (ALF) is a fulminant clinical syndrome characterized by extensive liver cell necrosis, resulting in severe liver dysfunction. This condition manifests with ascites, coagulation abnormalities, and rapid progression to complications such as hepatorenal syndrome, hepatic encephalopathy, and multiorgan failure in patients without prior liver disease [1, 2]. ALF poses a significant threat to human life, with a mortality rate of 60%–80% [3], and its incidence continues to rise annually. For instance, in the United States, the incidence of ALF has ranged from one case per million individuals annually to ~2000–3000 cases per year [1], with much higher rates observed in developing countries [4]. The etiology of ALF varies by region, with viral hepatitis being prevalent in the Asia-Pacific region, while acetaminophen poisoning and other drug-induced liver injuries are more common in Western countries [5–8]. Current treatment options for ALF include addressing the underlying cause, providing general supportive care, managing extrahepatic organ failure, utilizing artificial liver support systems, and performing orthotopic liver transplantation (OLT) [9–12]. Since 1983, OLT has been considered the most effective treatment for ALF [13], significantly improving patient survival rates [14, 15]. However, the availability of suitable donors, the inherent risks, the high costs associated with OLT, and the need for lifelong immunosuppressive therapy limit its widespread application [16–18]. Additionally, as the incidence of ALF continues to rise, the gap between patients in need of transplants and the availability of donor organs is growing [6, 19], highlighting the need for alternative therapeutic interventions. Recent research suggests that immunotherapy targeting macrophages may offer a promising alternative to treating ALF [20, 21].

As the first line of defense against environmental changes and damage, the innate immune system responds significantly faster than the adaptive immune response. This rapid response is particularly crucial in ALF, where the host has little time to activate an effective adaptive immune response [22]. Thus, the innate immune system may play a more crucial role than the adaptive immune system in the progression of ALF. Among the components of the liver's innate immune system, Kupffer cells (KCs) account for ~80%–90% of the tissue-resident macrophages in the liver [23–26]. Hepatic macrophages are key mediators in hepatocyte injury and are essential in regulating the initiation, amplification, and resolution of inflammatory responses, thereby playing a pivotal role in the onset and progression of ALF [27].

In response to various stimuli within the microenvironment, macrophages can be activated and reprogramed into different functional subtypes, typically polarizing toward classical (M1) or alternative (M2) activation. These subtypes are not fixed states but exist within a dynamic continuum, allowing for interconversion to meet changing demands and conditions of the environment [28]. M1 macrophages are pro-inflammatory and play a key role in pathogen defense, while M2 macrophages exhibit anti-inflammatory properties and promote tissue repair [29].

In the pathogenesis of acute liver injury, M1 macrophages predominantly secrete pro-inflammatory factors, while M2 macrophages primarily produce anti-inflammatory factors. By balancing these pro-inflammatory and anti-inflammatory agents through the suppression of M1 macrophage activation and the promotion of M2 macrophage activation, liver damage can be effectively minimized, thereby enhancing tissue repair in ALF [30–32]. Moreover, studies [33] have demonstrated that macrophages can dynamically alter their metabolic patterns and cellular functions in response to various environmental stimuli. This process involves changes in metabolic enzymes, metabolites, and pathways, enabling macrophages to obtain the necessary energy and metabolic intermediates for biosynthesis and cellular functions. The interplay between immune cell activation and energy metabolism is closely interlinked, where immune cells undergo metabolic changes following stimulation and activation, and the regulation of cellular energy metabolism impacts the immune response [34, 35]. Immune cells rely on six main metabolic pathways [36]: glycolysis, tricarboxylic acid (TCA) cycle, pentose phosphate pathway (PPP), fatty acid oxidation (FAO), fatty acid synthesis (FAS), and amino acid metabolism. The M1 macrophages predominantly derive their energy from glycolysis and the PPP, while the M2 macrophages depend on oxidative phosphorylation (OXPHOS) and FAO [37, 38].

This article explores the impact of glucose metabolism reprograming on the polarization of liver macrophages in ALF from a metabolic immunology perspective. Additionally, we summarize several traditional Chinese medicine (TCM) monomers that effectively regulate macrophage metabolism and reshape their polarization states. This article aimed to provide insights into potential therapeutic strategies for ALF.

## 2. Hepatic Macrophages and Their Role in ALF

### 2.1. Origin of Macrophages

Macrophages, first identified by Ilya Metchnikoff in the late 19th century [39], function as vital immune “sentinels” within the body. They bridge innate and adaptive immunity and play crucial roles in host defense, maintaining tissue integrity, and combating invading pathogens [40, 41]. Hepatic macrophages represent the largest population of innate immune cells in the liver, essential for sustaining overall body homeostasis and immune tolerance [42]. Studies [43–45] have demonstrated that the source of macrophages in the liver is heterogeneous, including liver-resident KCs, monocyte-derived macrophages (MDMs), liver capsular macrophages (LCMs), and splenic macrophages.

### 2.2. Polarization of Hepatic Macrophages

Macrophage polarization refers to the process by which macrophages are activated and differentiate into various subtypes in response to changes in the microenvironment, triggered by factors such as pathogenic microorganisms, inflammatory responses, cytokines, and certain physicochemical stimuli [46]. Macrophages exhibit high plasticity [47] and can polarize into different phenotypes when stimulated by pathogen-associated molecular patterns (PAMPs) or damage-associated molecular patterns (DAMPs) [48]. The M1 and M2 subtypes, analogous to the Th1 and Th2 classifications, are widely recognized [49]. However, the M1/M2 dichotomy represents only the two extremes of the macrophage activation spectrum. In reality, macrophages can exist in multiple activation states [50–52]. Hepatic macrophages can be activated by various agents, including interferon *γ* (IFN-*γ*), lipopolysaccharide (LPS), granulocyte-macrophage colony-stimulating factor (GM-CSF), or tumor necrosis factor (TNF), leading to their differentiation into classically activated M1 macrophages [31, 32]. M1 macrophages possess a strong antigen-presenting capacity and produce large amounts of pro-inflammatory cytokines such as IL-1*β*, TNF-*α*, IL-6, and IL-12. They also release nitric oxide (NO), reactive oxygen species (ROS), and reactive nitrogen species (RNS). Additionally, M1 macrophages secrete various chemokines that recruit other immune cells into the liver tissues, thereby promoting pro-inflammatory responses, pathogenic microbial clearance, and antitumor effects [29, 31, 32]. Hepatic macrophages can also polarize into M2 macrophages under specific stimuli, which can further be subdivided into four subtypes: M2a, M2b, M2c, and M2d [31, 32].

**Figure 1:**
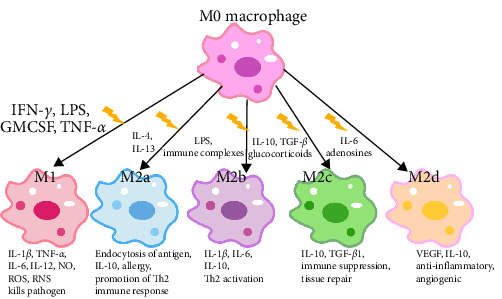
Polarization and functional effects of activated macrophages. GM-CSF, granulocyte-macrophage colony-stimulating factor; IFN-*γ*, interferon-*γ*; IL, interleukin; LPS, lipopolysaccharides; NO, nitric oxide; RNS, reactive nitrogen species; ROS, reactive oxygen species; TGF-*β*, transforming growth factor *β*; TNF-*α*, tumor necrosis factor-*α*; VEGF, vascular endothelial growth factor.

M2a macrophages, induced by IL-4 and IL-13, are involved in wound healing. They upregulate the mannose receptor (MR), secrete profibrotic factors, and contribute to tissue repair. M2b macrophages, induced by immune complexes and LPS, express high levels of IL-1*β*, IL-6, TNF-*α*, and IL-10. They secrete pro-inflammatory and anti-inflammatory cytokines, playing roles in protective and inflammatory processes. M2c macrophages, activated by IL-10, TGF-*β*, and glucocorticoids, inhibit inflammation, promote tissue repair, induce regulatory T cells, and engage in the engulfment of apoptotic cells. M2d macrophages, stimulated by IL-6, TLR ligands, and adenosine, upregulate vascular endothelial growth factor (VEGF) and IL-10. They share phenotypic and functional similarities with tumor-associated macrophages, thereby promoting angiogenesis and tumor cell metastasis [31, 32, 53] (Figure 1).

### 2.3. Role of Hepatic Macrophages in ALF

The current consensus is that the “endotoxin-macrophage-cytokine storm” is the core pathogenic mechanism of liver failure, with immune damage being the initiating factor, particularly in the early stages of the condition [54, 55]. Endotoxin, chemically known as LPS, is recognized by Toll-like receptor 4 (TLR4) [56], which is widely expressed on the surface of macrophages [57]. As key components of the innate immune system, macrophages play essential roles in microbial elimination, immune regulation, and tissue repair [50]. Hepatic macrophages, as first-line immune responders, significantly influence the progression of liver disease [22]. They are crucial for maintaining liver homeostasis and are actively involved in the process of acute and chronic liver injury and repair [58, 59]. Hepatocyte damage or death triggers the release of DAMPs, which in turn stimulate KCs and recruit MDMs to release of pro-inflammatory cytokines and chemokines [60]. KCs and MDMs can rapidly adjust their polarization states in response to local stimuli, influencing the progression and resolution of ALF [31]. The M1 and M2 phenotypes of macrophages typically play opposing roles in disease regulation and maintaining a balance between them is crucial for resolving inflammation and preserving tissue homeostasis [51, 61]. In the early stages of ALF, activated hepatic macrophages, particularly M1 macrophages, significantly increase, releasing pro-inflammatory factors that exacerbate liver injury. At this point, inducing M2 macrophages can counteract the pro-inflammatory effects of M1 macrophages, exerting anti-inflammatory, reparative, and immunomodulatory functions [21, 62]. However, in the later stages of ALF, excessive activation of M2 macrophages may lead to an exaggerated anti-inflammatory response, resulting in the deactivation of hepatic monocytes/macrophages. The potential treatment options in these cases include plasma exchange to remove IL-10 and secretory leukocyte protease inhibitors and restore immune balance [21, 62]. Additionally, studies have confirmed the alternation and imbalance between systemic inflammatory response syndrome and compensatory anti-inflammatory response syndrome during the course of ALF, leading to immune dysregulation [63]. Therefore, identifying the immune status in ALF, determining the optimal timing for intervention, and redirecting macrophage functions to reduce liver damage, facilitate tissue repair, and promote liver regeneration are critical in the field of immunomodulatory therapy for ALF.

## 3. Interplay Between Glucose Metabolism and Hepatic Macrophages

All cells require energy metabolism to produce ATP and metabolic intermediates, which are essential for their survival, proliferation, and differentiation [64]. Recent studies [38, 65] have shown that cellular energy metabolism can influence the polarization of macrophages, thereby regulating their functional roles. The metabolic profile of M1 macrophages is primarily characterized by enhanced glycolysis, PPP, and FAS, with a concurrent reduction in the activity of the TCA and OXPHOS pathways. In contrast, M2 macrophages exhibit enhanced OXPHOS, FAO, and glutamine metabolism [66, 67]. However, depending on the stimulus, OXPHOS may also be upregulated to promote inflammation. Research has shown that OXPHOS can generate ROS through Complex I [68], and excessive ROS production can lead to tissue damage and chronic inflammation [69]. Inhibiting Complex I has been found to reduce the activity of the IFN-*γ* receptor [70]. Furthermore, studies have revealed that prolonged exposure of macrophages to degradation products of polylactic acid results in an increase in both OXPHOS and glycolysis. This elevation results in heightened expression of proteins such as IL-6, MCP-1, TNF-*α*, and IL-1*β*, thereby enhancing the production of pro-inflammatory cytokines [71].

**Figure 2:**
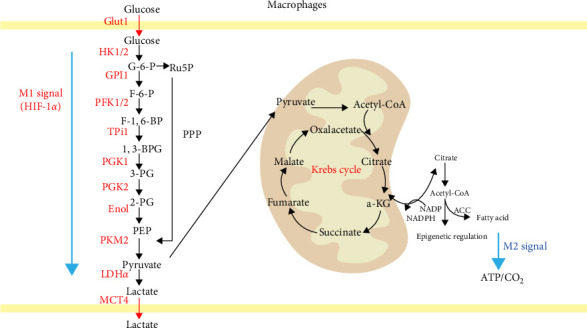
Relationship between macrophage polarization and glucose metabolism. 1,3-BPG, 1,3-diphosphoglycerate; 2-PG, 2-phosphoglycerate; 3-PG, 3-phosphoglycerate; ACC, acetyl-coenzyme A carboxylase; Acetyl-CoA, acetyl-coenzyme A; a-KG, a-ketoglutarate; ENO1, alpha-enolase; F-1,6-BP, fructose 1,6-bisphosphate; F-6-P, fructose-6-phosphate; G-6-P, glucose-6-phosphate; GLUT1, glucose transporter 1; GPI, glucose phosphate isomerase; HK1/2, hexose kinase 1/2; LDH a, lactate dehydrogenase α; MCT4, monocarboxylate transporter protein 4; PEP, phosphoenolpyruvate; PFK1/2, phosphofructokinase; PGK1, phosphoglycerate kinase 1; PGK2, phosphoglycerate kinase 2; PKM2, pyruvate kinase 2; Ru5p, ribulose 5-phosphate; TPI1, triosephosphate isomerase 1.

Under steady-state conditions, cells primarily rely on the OXPHOS pathway for energy production, with each glucose molecule producing 36 molecules of ATP through the TCA cycle. However, in a hypoxic environment, pyruvate produced by glycolytic metabolism is converted into lactate instead of entering the TCA cycle, yielding only 2 ATP molecules in this process [72, 73]. In the early 20th century, Warburg, Wind, and Negelein [74] discovered that tumor cells exhibit active glycolytic metabolism and consumed large amounts of glucose even in the presence of sufficient oxygen, a phenomenon known as the “Warburg effect.” Although glycolysis is less efficient in ATP production and requires increased glucose consumption, its rate of glucose metabolism is 10–100 times faster than that of OXPHOS [75, 76]. However, the study by Dengler et al. [77] has demonstrated that excessive aerobic glycolysis can lead to cell death. In 1970, Hard [78] identified a Warburg effect similar to that of tumor cells in LPS-activated M1 macrophages. During this process, M1 macrophages primarily rely on aerobic glycolysis for energy production, resulting in reduced OXPHOS metabolism, increased glucose consumption, and lactate synthesis [79]. Additionally, hypoxia-inducible factor 1*α* (HIF-1*α*) upregulates the expression of glycolysis-related enzymes such as glucose transporter 1 (GLUT1), hexokinase-2 (HK2), phosphate fructose kinase 1/2 (PFK-1/2), and pyruvate kinase isozyme M2 (PKM2) [80, 81] (Figure 2). Additionally, studies [82, 83] have demonstrated that modulating macrophage energy metabolism can regulate macrophage polarization. Specifically, inhibiting glycolysis while promoting OXPHOS and FAO suppresses M1 macrophage activation and enhances M2 macrophage activation.

Moreover, with the advancement of research in metabolic reprograming, it has been demonstrated that intermediate metabolites generated through these processes, such as acetyl-coenzyme A (acetyl-CoA), *α*-ketoglutarate (*α*-KG), and NAD+, serve as substrates or cofactors that play critical roles in the epigenetic modification of tumors [84]. Similarly, the involvement of these metabolites in regulating macrophage polarization is significant. Studies have revealed that citrate possesses an influential position in maintaining the macrophage inflammatory response [85]. In LPS-activated macrophages, the upregulation of glycolytic genes occurs when citrate carriers export citrate from mitochondria via histone acetylation [86]. Itaconate and its derivatives commonly inhibit macrophage activation. They not only suppress pro-inflammatory M1 macrophages but also prevent M2 polarization by inhibiting JAK1 phosphorylation [87]. The research results may seem contradictory, as itaconic acid primarily exerts anti-inflammatory effects. Certain studies have shown that it limits inflammatory responses through the activation of the Nrf2 pathway [88]. Additionally, succinate can upregulate the transcription of metastasis-associated genes via HIF-1*α* [89] and enhance the production of the pro-inflammatory cytokine IL-1*β* through HIF-1*α* induction [90]. *α*-KG is essential for M2 macrophage activation, including involvement in FAO and Jmjd3-dependent epigenetic reprograming of M2 genes [91].

## 4. The Regulatory Mechanisms of Macrophage Polarization

M1/M2 macrophage activation is a highly regulated process [92], with these macrophage subsets exhibiting distinct metabolic profiles [85]. As previously discussed [82, 83], macrophage activation involves metabolic reprograming, and modulating macrophage energy metabolism can influence their polarization state. It has been demonstrated that the metabolic profile of macrophage polarization is regulated by multiple signaling pathways [81]. In this review, we focus on four principal signaling pathways: the phosphatidyl-inositol 3-kinase/protein kinase B (PI3K/AKT) pathway, the mammalian target of rapamycin (mTOR)/HIF-1*α* signaling pathway, the nuclear factor-*κ*B (NF-*κ*B) pathway, and the AMP-activated protein kinase (AMPK) signaling pathway. These signaling pathways are pivotal not only for macrophage polarization but also for the regulation of macrophage metabolism.

### 4.1. PI3K/Akt Signaling Pathway

PI3K signaling is crucial for regulating cell growth, proliferation, metabolism, inflammation, survival, motility, and tumor progression [93, 94]. Upon activation, PI3K phosphorylates Akt, which in turn activates p-Akt. Activated p-Akt can further activate mTOR and regulate HIF-1*α*, playing a central role in glycolysis, cancer metabolism, and cancer cell proliferation [95–97]. The PI3K/Akt signaling pathway regulates the survival, migration, and proliferation of macrophages, and it coordinates their responses to various metabolic and inflammatory signals [98]. Studies [98–100] have demonstrated that Akt1 promotes macrophage polarization toward the M2 phenotype while inhibiting polarization toward the M1 phenotype. Conversely, Akt2 stimulates macrophage polarization towards the M1 phenotype and inhibits polarization towards the M2 phenotype.

### 4.2. mTOR/HIF-1*α* Signaling Pathway

mTOR is a serine/threonine kinase that plays a crucial role in regulating cell metabolism, including growth, proliferation, and survival and is pivotal in macrophage polarization [101, 102]. It functions through two distinct protein complexes: mTOR complex 1 (mTORC1) and mTOR complex 2 (mTORC2) [103]. Wu et al. [82] reported that activation of mTORC1 induces glycolysis through a HIF-1*α*-dependent mechanism, promoting M1-polarization, while mTORC2 activation stimulates peroxisome proliferator-activated receptor *γ* (PPAR *γ*), enhancing FAO and promoting M2 polarization. Furthermore, Byles et al. [102] found that the deletion of tuberous sclerosis complex 1 (TSC1), which leads to mTORC1 overexpression, results in enhanced M1 polarization and impaired M2 polarization. Recent studies [104] have confirmed that mTOR-induced glycolysis is mediated through the activation of HIF-1*α* and the stimulation of glycolytic enzymes. Conversely, the mTOR inhibitor rapamycin has been shown to inhibit HIF-1*α* expression [105]. HIF-1*α*, an essential regulator of aerobic glycolysis, facilitates the transition from OXPHOS to glycolysis, thus promoting M1 macrophage activation [90, 106–108]. Specifically, HIF-1*α* transcriptionally activates genes involved in oxygen homeostasis and metabolic activation [109]. Additionally, it upregulates various glycolysis-related proteins, such as GLUT1, hexokinase 3, and 6-phosphofructo-2-kinase, which increase glycolysis levels and influence macrophage polarization [90, 110]. In a mouse model of ALF, Cai et al. [111] demonstrated that downregulation of HIF-1*α* expression inhibits glycolysis, thereby reducing macrophage infiltration and M1 polarization in liver tissue.

### 4.3. NF-*κ*B Signaling Pathway

The NF-*κ*B signaling pathway, a well-known pro-inflammatory pathway, plays a central role in regulating gene transcription during immune and inflammatory responses [112, 113]. NF-*κ*B is intricately linked to metabolic processes, influencing the balance between glycolysis and mitochondrial respiration, which is essential for managing the energy metabolism network [114]. Specifically, the TLR4/NF-*κ*B signaling pathway is involved in inducing acute liver injury under LPS stimulation [115]. TLR4 is a signal-transducing transmembrane receptor located on the cell membrane, and NF-*κ*B is an important molecule downstream of the TLR4 signaling pathway [116]. After TLR4 specifically recognizes LPS and binds to it, it relies on myeloid differentiation protein 88. Upon activation, NF-*κ*B dissociates from its inhibitor, translocates to the nucleus, and binds to the promoter regions of target genes, thereby regulating their expression and promoting the secretion of inflammatory factors such as TNF-*α*, IL-1*β*, and IL-6 [117]. NF-*κ*B is also crucial in the metabolism and polarization of M1 macrophages [112, 113]. Activation of NF-*κ*B initiates the transcription of pro-inflammatory genes and facilitates the transition from M2 to M1 macrophages [118]. Studies [119] have shown that upon activation by LPS, the NF-*κ*B signaling pathway increases GLUT6 expression, thereby enhancing glycolysis and the secretion of inflammatory mediators in M1 macrophages. Additionally, NF-*κ*B acts as a direct regulator of HIF-1*α* expression during inflammation and hypoxia. NF-*κ*B activation upregulates HIF-1*α* transcription, which promotes macrophage glycolysis and M1 polarization, thus enhancing the host's immune defense responses. Conversely, HIF-1*α* provides a feedback mechanism to inhibit NF-*κ*B transcriptional activity both in vivo and in vitro during inflammatory states, thereby preventing excessive and destructive pro-inflammatory responses [120, 121].

### 4.4. AMPK Signaling Pathway

AMPK, a protein kinase that monitors changes in energy molecules, is involved in signal transduction of multiple signaling pathways and is essential for maintaining cellular energy homeostasis [122]. It consists of three subunits: *α*, *β*, and *γ*, with the *γ* subunit containing binding sites for AMP, ADP, and ATP, enabling it to sense the AMP/ATP ratio. AMPK is activated when there is an increase in the intracellular AMP/ATP ratio when calcium ion flux rises. Once activated, AMPK phosphorylates pivotal proteins through various pathways, promoting catabolism to generate more ATP and inhibiting anabolism to reduce ATP consumption [123–125]. In terms of macrophage polarization, AMPK is considered a negative regulator of M1 macrophage activation induced by LPS [126]. Sag et al. [127] found that stimulation of macrophages with anti-inflammatory factors like IL-10 and TGF-*β* resulted in the activation of AMPK, whereas stimulation with pro-inflammatory factors like LPS results in AMPK inactivation. Suppression of AMPK activity, achieved through siRNA knockdown or introduction of a dominant-negative mutant, it is demonstrated that suppressing AMPK activity increased the synthesis of TNF-*α* and IL-6 while decreasing the production of IL-10. Conversely, constitutively active AMPK*α*1 decreased LPS-induced TNF-*α* and IL-6 production while increasing IL-10 levels, indicating that AMPK activation inversely regulates the macrophage inflammatory signaling pathway. Furthermore, studies [128] have demonstrated that AMPK activation antagonizes the Warburg effect by inhibiting HIF-1*α* and its associated glycolytic effectors. Importantly, AMPK and mTOR signaling pathways are interconnected and exhibit opposing effects on nutrient sensing, energy availability, and cell growth regulation [129, 130]. They function as balance-regulating switches in the M1/M2 macrophage transformation, detecting changes in cell metabolites and promoting signals for M1 or M2 activation, thus maintaining the equilibrium between pro-inflammatory and anti-inflammatory responses [92].

## 5. Modulation of Glucose Metabolism Reprograming by Chinese Medicine Affects Hepatic Macrophage Polarization to Intervene in ALF

### 5.1. Melittin

Melittin, the principal bioactive component of bee venom, constitutes ~40%–50% of bee venom's total dry weight [131]. Despite its inherent toxicity, recent studies have highlighted its potential benefits, including anti-inflammatory, anticancer, antibacterial, and antiviral properties [132]. Naji et al. [133] demonstrated that Melittin could mitigate drug-induced liver injury induced by isoniazid and rifampicin in rats. Their findings indicated that Melittin improved biochemical indicators and liver histopathology, suggesting its potential in preventing antituberculosis drug-induced ALF. Similarly, Park et al. [134] observed in a mouse model of liver failure induced by LPS/D-GalN that Melittin reduced the release of inflammatory cellular factors and prevented hepatocyte apoptosis, likely through the inhibition of NF-*κ*B activation. These studies provide evidence supporting Melittin's protective effects against acute liver injury. Moreover, Fan et al. [135] investigated the mechanism from an immunometabolism perspective, elucidating the effects of Melittin. Their study indicated that in a mouse model of ALF induced by LPS/D-GalN, the expression of PKM2 and HIF-1*α* was upregulated in liver tissues. Treatment with bee venom significantly reduced the levels of PKM2 and HIF-1*α*. LPS-induced activation elevated the glycolytic rate and glycolytic product levels of RAW264.7 cells, but melittin intervention inhibited PKM2 and induced a shift in energy metabolism, resulting in an anti-inflammatory effect. Further mechanistic analysis suggests that the Akt/mTOR/PKM2/HIF-1*α* signaling pathway is upregulated during ALF progression. However, this signaling pathway's activation was suppressed by Melittin. Additionally, Melittin inhibited PKM2 activity and mitigated the PKM2-mediated Warburg effect, thereby controlling the inflammatory response associated with macrophage activation.

### 5.2. Quercetin

Quercetin, a flavonoid found in various dietary sources such as vegetables, fruits, nuts, and tea, possesses notable anti-inflammatory, antioxidant, and anticancer properties [136, 137]. Previous studies [138] indicate that quercetin exhibits anti-inflammatory effects by regulating immune cell activation, inhibiting the release of pro-inflammatory factors, and down-regulating inflammatory gene transcription. These effects involve the modulation of several signaling pathways, including NF-*κ*B, mitogen-activated protein kinases (MAPK), and arachidonic acid (AA). Mendes et al. [139] explored the metabolic reprograming of glucose in macrophages derived from THP-1 by treated with LPS + IFN-*γ*. Their findings revealed that quercetin inhibited glycolysis and promoted the TCA cycle, demonstrating superior pharmacological efficacy compared to other flavonoids in reversing macrophage glucose metabolism reprograming. In another study, Tsai et al. [140] pretreated RAW264.7 cells and adult mouse microglia with quercetin before stimulation with LPS. The results demonstrated that quercetin significantly reduced the expression of M1 macrophage markers such as IL-6, IL-1*β*, and TNF-*α*. It also reduced the release of chemokines linked to M1 polarization and suppressed the production of NO. Additionally, quercetin inhibited the expression of inducible NO synthase (iNOS) and cyclooxygenase 2 (COX-2) while enhancing the expression of the M2 macrophage marker IL-10 through activation of AMPK and Akt signaling pathways when directly applied to RAW264.7 and adult mouse microglia cells. Additionally, quercetin upregulated the endogenous antioxidant system and reduced ROS production. Moreover, glycyrrhizin-mediated liver-targeted alginate nanogels have been developed to deliver quercetin directly to the liver. This novel pharmaceutical formulation demonstrated promising effects in treating acute liver injury, as evidenced by improved biochemical indexes, reduced peroxide parameters, and downregulation of TNF-*α*, IL-6, iNOS, and monocyte chemotactic protein-1 (MCP-1) [141].

### 5.3. Salvianolic Acid B

Salvianolic acid B, a prominent active compound derived from *Salvia miltiorrhiza* root, exhibits a spectrum of pharmacological effects, including antioxidant, anti-inflammatory, antiapoptotic, and antifibrotic effects [142]. Studies [143, 144] have demonstrated its hepatoprotective and antifibrotic effects on the liver, including its ability to inhibit hepatocyte apoptosis and reduce oxidative stress-induced hepatocyte damage. Huang et al. [145] further confirmed the beneficial impact of salvianolic acid B on cellular energy metabolism by upregulating PPAR*α* expression and promoting the phosphorylation of AMPK and acetyl-CoA carboxylase (ACC) in liver tissues. Moreover, Zhao et al. [146] found that salvianolic acid B could shift macrophage polarization from pro-inflammatory M1-type to anti-inflammatory M2 macrophages by inhibiting mTORC1-induced glycolysis. Additionally, Wei et al. [147] elucidated the ability of salvianolic acid B to inhibit glycolysis and regulate abnormal glucose metabolism through modulation of the PI3K/AKT/HIF-1*α* signaling pathway.

### 5.4. *Lycium barbarum* Polysaccharides (LBP)

LBP, the primary bioactive constituent of wolfberry, possesses antioxidative, antitumor, immune-modulating, neuroprotective, and hepatoprotective effects [148]. Ding et al. [149] used LPS to induce the polarization of RAW264.7 cells toward the M1 phenotype, noting an upregulation of PKM2 and HIF-1*α* expression, increased glycolysis, and elevated secretion of inflammatory mediators such as IL-1*β* and TNF-*α*. However, upon LBP intervention, macrophage polarization toward the M1 phenotype was inhibited, accompanied by reduced expression of PKM2 and HIF-1*α*, suppressed glycolysis, and decreased secretion of inflammatory mediators. Remarkably, the therapeutic effects of LBP closely resembled those achieved through PKM2 knockdown and could be reversed by PKM2 overexpression, leading to the hypothesis that LBP suppresses glycolysis and modulates macrophage polarization by downregulating PKM2. Liu et al. [150] conducted a similar study, demonstrating that LBP could attenuate the expression levels of inflammatory mediators (TNF-*α*, IL-1*β*, and IL-6) and NO by regulating macrophage polarization and NF-*κ*B translocation. Notably, these effects were primarily mediated through the inhibition of TLR4 and the NF-*κ*B signaling pathways.

### 5.5. Cassiaside C

*Cassia obtusifolia*, the dried seeds of cassia plant from the Leguminosae family, possesses various therapeutic properties, including anti-inflammatory, hepatoprotective, antidiarrheal, antidiabetic, antibacterial, and neuroprotective activities. It has been used clinically to treat acute liver injury, constipation, Alzheimer's disease, hypertension, and hyperlipidemia [151]. Among its primary active components, Cassiaside C, a naphthopyrone compound, has garnered significant attention [152]. Kim et al. [83] found that Cassiaside C could inhibit LPS/IFN-*γ*-induced polarization of RAW264.7 and peritoneal macrophages to the M1 phenotype by downregulating the PI3K/AKT/mTORC1 signaling pathway, thereby reducing the transcription and secretion of pro-inflammatory factors such as TNF-*α*, IL-1*β*, and IL-6. Furthermore, Cassiaside C was shown to decrease glycolysis levels and lactate production.

### 5.6. Cynaroside

Cynaroside, a flavonoid isolated from the *Bidens parviflora* Willd plant [153], exhibits anti-inflammatory, antioxidant, and antitumor biological effects [154]. It has been demonstrated to hinder glycolysis by inhibiting HK2 [155]. In a study by Pei et al. [156], Cynaroside was observed to suppress liver PKM2 expression and nuclear translocation. This suppression led to a reduction in PKM2 binding to HIF-1*α*, inhibition of glycolysis-related enzymes, promotion of the transformation from M1 to M2 macrophages, attenuation of pro-inflammatory factor release, and, ultimately, positive anti-inflammatory effects.

### 5.7. Ginsenoside Rg3

Ginsenoside Rg3 is a member of the ginsenoside family of saponins, which, according to modern pharmacological studies, exhibits a range of biological activities, including anti-inflammatory, hepatoprotective, antiallergic, and antitumor effects [157]. Ginsenoside Rg3 has been shown to inhibit M1 macrophage polarization and induce M2 macrophage polarization, leading to anti-inflammatory effects [158]. Through proteomics and metabolomics analysis, Ni et al. [159] found that ginsenoside Rg3 could activate AMPK to inhibit glycolysis and significantly modulate various metabolites such as pyruvate, acetyl-CoA, isocitrate, and succinate during glycolysis and the TCA cycles. Ginsenoside Rg3 [160, 161] also accelerates the resolution of inflammation by inducing M2 macrophage polarization, which may be dependent on the regulation of the NF-*κ*B pathway. The aforementioned Chinese herbal monomers are summarized in Table 1.

## 6. Discussion

**Figure 3:**
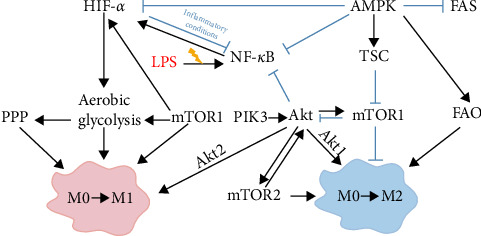
Crosstalk between regulatory signaling pathways in macrophage polarization. Macrophages can polarize into M1 or M2 phenotype upon LPS stimulation. The signaling pathways governing this polarization are intricate and interdependent, involving PI3K/Akt, mTOR/HIF-1*α*, NF-*κ*B, and AMPK, among others [90, 98, 100, 102, 106–108, 120, 121, 162, 164–181]. AMPK, AMP-activated protein kinase; FAO, fatty acid oxidation; FAS, fatty acid synthesis; HIF-1*α*, hypoxia-inducible factor-1*α*; LPS, lipopolysaccharide; M0, unpolarized macrophages; M1, M1-type macrophages; M2, M2-type macrophages; mTORC1, mTOR complex 1; NF-*κ*B, nuclear transcription factor-*κ*B; PI3K/AKT, phosphatidylinositol 3/protein kinase B; PPP, pentose phosphate pathway; TSC, tuberous sclerosis complex.

ALF is a rare yet life-threatening condition characterized by complex etiologies and high mortality rates [163]. While OLT remains the primary treatment for ALF, its clinical application is limited [11]. Hence, there is an urgent need to explore and develop novel drugs with innovative mechanisms of action for ALF. It represents a critical clinical challenge requiring immediate attention. Macrophages, as vital components of the innate immune system, are pivotal in the initiation, progression, and resolution of ALF [27]. During different stages of ALF, macrophages can polarize into either the classically activated M1 pro-inflammatory phenotype or the alternatively activated M2 anti-inflammatory phenotype, each exerting distinct biological functions [62]. Research indicates that promoting the polarization of macrophages from M1 to the M2 phenotype can effectively alleviate liver damage and promote liver tissue repair [31]. Current research on macrophage polarization, based on immunometabolism, mainly focuses on key signaling pathways such as PI3K/Akt, mTOR/HIF-1*α*, NF-*κ*B, and AMPK. These pathways play vital roles in the metabolic regulation and polarization of macrophages and are closely interrelated, as depicted in Figure 3.

Modern pharmacological studies indicate that TCM, such as Cassiaside C [83], Melittin [135], LBP [149], and Cynaroside [156], can effectively regulate the polarization of M1/M2 macrophages in ALF through glucose metabolism reprograming, thereby exerting anti-inflammatory and hepatoprotective effects. It is important to note that current research on glucose metabolic reprograming by TCM primarily focuses on isolated components or monomers of Chinese herbal compounds. This approach is crucial for elucidating the material basis and mechanisms of action of TCM. However, Chinese herbal formulations, which are the primary form used in clinical practice, inherently possess unique characteristics of multiple components, targets, and effects. Leveraging these characteristics can offer the advantage of integrated therapeutic effects. Therefore, it is essential not only to explore individual components of TCM but also to intensify and deepen research on Chinese herbal formulations.

It is worth noting that although many reports highlight the use of TCM in treating ALF, there is a lack of studies focusing on metabolic immunology and metabolic reprograming. Future experimental designs that incorporate perspectives from metabolic immunology and metabolic reprograming hold significant promise for advancing herbal medicine in ALF treatment. This novel and promising approach can be implemented through several key aspects:

1. Preliminary screening of target components: Methods such as serum medicinal chemistry, “spectrum-effect” correlation analysis, in vitro cell models, near-infrared spectroscopy, TCM chromatographic fingerprinting, and real-time cell-based assays, and computer virtual screening can be employed to identify potential target components. Subsequently, the pharmacodynamic substance basis of TCM can be elucidated using target component knockout/knock-in technology [182]. Furthermore, innovative techniques like mass spectrometry chromatography [183], chromatography [184], small molecule probe [185], network pharmacology [186, 187], and bioinformatics [188] can be used to determine the specific targets of TCM in the treatment of ALF.
2. Structural modification and optimization: The structural modification and optimization of active ingredients, along with the use of nanoparticle technology, can enhance drug targeting. For instance, Zhao et al. [141] successfully delivered the antioxidant quercetin to the liver for ALF by employing glycyrrhizin-mediated liver-targeted alginate nanogels. This approach significantly improves drug targeting and bioavailability, demonstrating the potential of exploring TCM resources in developing new drugs for ALF.
3. Systematic and networked integration analysis: A comprehensive analysis of anti-ALF active components, mechanisms of action, and drug metabolism of TCM can be conducted using multirecombinant technologies such as genomics, transcriptomics, proteomics, and metabolomics. Notably, Xu et al. [189] have completed the whole genome sequencing of *Salvia miltiorrhiza*, providing a crucial genetic background for understanding the biosynthesis and molecular regulatory mechanisms of its main pharmacologically active components, marking the beginning of the genomics era of TCM.
4. Construction of specific disease models: Disease models can be constructed based on TCM patterns of evidence. For instance, Fu et al. [190] used serum from patients with liver depression and spleen deficiency syndrome to induce Hep G2 cells, building a cellular model that reflects the biological basis of the syndrome to a certain extent.
5. High-quality clinical trials: High-quality clinical trials related to TCM should be designed and conducted to ensure safety and efficacy. These trials should provide high-level evidence-based data to support clinical decision-making.

In conclusion, targeting hepatic macrophages as a therapeutic strategy for treating ALF holds considerable potential. Encouraging the shift of hepatic macrophages from the M1 to M2 phenotype appears promising in reducing liver damage and fostering liver regeneration. However, the polarization state of liver macrophages is intricately intertwined with the reprograming of glucose metabolism. Transitioning the energy metabolism profile from glycolysis to OXPHOS can robustly regulate macrophage polarization, thereby exerting anti-inflammatory and hepatoprotective effects. Additionally, TCMs have shown favorable results in modulating the energy metabolism of liver macrophages. Therefore, our future direction will involve the design and execution of rigorous and comprehensive investigations at various levels, including biochemical, cellular, animal, and human studies, to delve further into this domain.

Figure 1: Macrophages can differentiate into classically activated M1 macrophages in response to stimuli such as IFN-*γ*, LPS, GM-CSF, or TNF. M1 macrophages secrete IL-1*β*, TNF-*α*, IL-6, IL-12, NO, ROS, and RNS, contributing to pro-inflammatory responses, pathogenic microbial clearance, and antitumor effects. M2a macrophages, induced by IL-4 and IL-13, secrete IL-10 and play a role in tissue repair. M2b macrophages, activated by immune complexes and LPS, exhibit high expression levels of IL-1*β*, IL-6, and IL-10, which are involved in protective and inflammatory responses. M2c macrophages, activated by IL-10, TGF-*β*, and glucocorticoids, express high levels of IL-10 and TGF-*β*1, contributing to the inhibition of inflammation and the promotion of tissue repair. M2d macrophages, activated by IL-6, TLR ligands, and adenosine, display upregulated VEGF and IL-10, with anti-inflammatory and angiogenic effects.

Figure 2: Macrophages with different polarization states exhibit significant differences in their glucose metabolism pathways. M1 macrophages primarily derive energy from glycolysis, with a disruption in the TCA cycle. Key enzymes involved in the energy metabolism of M1 macrophages include Glut1, HK1/2, GPI1, PFK1/2, TPI1, PGK1, PGK2, Eno1, PKM2, and LDH*α*. This metabolic process results in the production of lactate, which is expelled from the cell by the MCT4 transporter. Some glucose also enters the PPP. In contrast, M2 macrophages rely predominantly rely on OXPHOS and FAO for energy. Pyruvate in M2 macrophages is metabolized through the TCA cycle (Krebs cycle), producing intermediates such as acetyl-CoA, citrate, and *α*-KG. These intermediates further contribute to FAS and epigenetic regulation, ultimately generating ATP and CO_2_.

## Figures and Tables

**Table 1:** Summary of Chinese medicine monomers.

Chinese herbal monomer	Biological activity	Mechanism	Outcome
Melittin	Anti-inflammatory, anticancer, antibacterial, antiviral	Inhibition of NF-*κ*B	Reduced the release of cellular inflammatory factors and inhibited hepatocyte apoptosis [134]
		Inhibition of PKM 2	Suppression of the Warburg effect [135]

Quercetin	Anti-inflammatory, antioxidant, and anticancer	Regulation of NF-*κ*B, MAPK, AA, etc.	Anti-inflammatory [138]
		Activation of AMPK, Akt	Increased expression of M2 macrophage marker (IL-10) [140]

Salvianolic acid B	Antioxidation, anti-inflammatory, antiapoptotic and antifibrotic, hepatoprotective	Inhibition of mTORC1	Inhibition of glycolysis and conversion of M1-type macrophages to M2-type macrophages [146]
		Modulation of PI3K/AKT/HIF-1*α*	Inhibited glycolysis and regulated abnormal glucose metabolism [147]

Lycium barbarum polysaccharides	Antioxidant, antitumor, immunomodulatory, neuroprotective, hepatoprotective	Inhibition of PKM2	Inhibited glycolysis and M1 polarization in macrophages [149]
		Inhibition of TLR4 and NF-*κ*B	Regulation of macrophage polarization, attenuation of inflammatory mediators, and NO expression [150]

Cassiaside C	Anti-inflammatory, liver protection, antidiarrhea, antidiabetes, antibacterial, and neuroprotective	Inhibition of PI3K/AKT/mTORC1	Inhibited glycolysis levels, reduced lactate production, inhibited M1 polarization in macrophages, and reduced the expression of pro-inflammatory cytokines [83]

Cynaroside	Anti-inflammatory, antioxidant, and anticancer	Inhibition of HK2	Inhibited glycolysis [155]
		Inhibition of PKM 2	Inhibited glycolysis-related enzymes, induced the transition of M1-type macrophages to M2-type macrophages, decreased the secretion of pro-inflammatory factors, and exerted anti-inflammatory effects [162]

Ginsenoside Rg3	Anti-inflammation, hepatoprotection, antiallergy, antitumor	Activation of AMPK	Inhibition of glycolysis [159]
		Inhibition of NF-*κ*B	M2 macrophages polarization, M2 macrophages polarization [160, 161]

## Data Availability

This article is a review study and does not contain any original data. All data and information cited in the text are derived from published literature and publicly available resources. Detailed references to these resources are listed in the article's references section. Due to the nature of this review, no additional dataset needs to be shared or stored.
